# The efficacy of dapagliflozin for type 1 diabetes: a meta-analysis of randomized controlled studies

**DOI:** 10.4314/ahs.v21i1.2

**Published:** 2021-03

**Authors:** Jian Ma, Yanhong Zhao, Huihui Fan, Jia Liu

**Affiliations:** 1 Department of endocrinology, Fenghua people's hospital, Ningbo, Zhejiang, China, 300061; 2 Obstetrics and Gynecology Department, Yuyao second people's hospital, NingBo, Zhejiang, China, 300061

**Keywords:** Dapagliflozin, type 1 diabetes, glycemic control, randomized controlled trials

## Abstract

**Introduction:**

The efficacy of dapagliflozin for type 1 diabetes remains controversial. We conduct a systematic review and meta-analysis to explore the treatment efficacy of dapagliflozin versus placebo in patients with type 1 diabetes.

**Methods:**

We have searched PubMed, EMbase, Web of science, EBSCO and Cochrane library databases through May 2019 for randomized controlled trials (RCTs) assessing the effect of dapagliflozin versus placebo for type 1 diabetes. This meta-analysis is performed using the random-effect model.

**Results:**

Six RCTs are included in the meta-analysis. Overall, compared with control group for type 1 diabetes, dapagliflozin treatment shows favorable impact on glycated hemoglobin HbA1c (standard mean difference SMD=-3.93; 95% confidence interval CI =-4.44 to -3.48; P<0.00001), HbA1c reduction of ≥0.5% (risk ratio RR=1.98; 95% CI=1.65 to 2.39; P<0.00001), and fasting plasma glucose FPG (SMD=-0.93; 95% CI=-1.77 to -0.10; P=0.03). There is no statistical difference of hypoglycemia (RR=1.09; 95% CI=0.66 to 1.79; P=0.75) or adverse events (RR=1.07; 95% CI=0.96 to 1.20; P=0.20) between two groups, but the incidence of ketone-related events is higher than those in control group (RR=0.28; 95% CI=3.96 to 11.52; P=0.01).

**Conclusions:**

Dapagliflozin treatment benefits to reduce HbA1c and FPG for type 1 diabetes.

## Introduction

It is estimated that less than one-third of patients with type 1 diabetes obtain optimal glycemic control 1, 2, and these patients may have increased mortality even after achieving targeted HbA1c levels 3–5. Insulin therapy is widely accepted as the mainstay of treatment, but may result in hypoglycemia, weight gain and cardiovascular events 6–9. These complications negatively affect the quality of life and increase the mortality 10, 11.

Sodium-glucose cotransporter 2 (SGLT2) inhibitors are the insulin-independent, glucose-dependent antihyperglycemic agents and demonstrate some potential in the treatment of type 1 diabetes 12–15. They were reported to result in weight loss and decreased glycemic variability in patients with type 1 diabetes. For instance, many studies revealed that dapagliflozin, an SGLT2 inhibitor held some promise in glycemic control for type 1 diabetes 16–19. In patients with inadequately controlled type 1 diabetes, dapagliflozin treatment was associated with significantly decreased HbA1c, body weight, total insulin dose and glycemic variability 20.

Current evidence is insufficient for routine clinical use of dapagliflozin for patients with type 1 diabetes. Recently, several studies have investigated the efficacy and safety of dapagliflozin for these patients, but the results are conflicting 17, 18, 21. This systematic review and meta-analysis of RCTs aims to assess the impact of dapagliflozin versus placebo on glycemic control for type 1 diabetes.

## Materials and methods

This systematic review and meta-analysis were performed based on the guidance of the Preferred Reporting Items for Systematic Reviews and Meta-analysis statement and Cochrane Handbook for Systematic Reviews of Interventions 22, 23. No ethical approval and patient consent were required because all analyses were based on previous published studies.

### Literature search and selection criteria

We have systematically searched several databases including PubMed, EMbase, Web of science, EBSCO, and the Cochrane library from inception to May 2019 with the following keywords: “dapagliflozin”, and “diabetes”. The reference lists of retrieved studies and relevant reviews were also hand-searched and the process above was performed repeatedly in order to include additional studies.

The inclusion criteria were presented as follows: (1) study design was RCT, (2) patients were diagnosed with type 1 diabetes, and (3) intervention treatments were dapagliflozin versus placebo.

### Data extraction and outcome measures

Some baseline information was extracted from the original studies, and they included first author, number of patients, age, body mass index (BMI), duration of disease, and detail methods in two groups. Data were extracted independently by two investigators, and discrepancies were resolved by consensus. We contacted the corresponding author to obtain the data when necessary.

The primary outcomes were glycated hemoglobin (HbA1c) and HbA1c reduction of ≥0.5%. Secondary outcomes included fasting plasma glucose (FPG), hypoglycemia, ketone-related events and/span>adverse events.

### Quality assessment in individual studies

The methodological quality of each RCT was assessed by the Jadad Scale which consisted of three evaluation elements: randomization (0–2 points), blinding (0–2 points), dropouts and withdrawals (0–1 points) 24. One point would be allocated to each element if they were conducted and mentioned appropriately in the original article. The score of Jadad Scale varied from 0 to 5 points. An article with Jadad score≤2 was considered to be of low quality. The study was thought to be of high quality if Jadad score≥3 25.

### Statistical analysis

We assessed standard mean difference (SMD) with 95% confidence interval (CI) for continuous outcomes (HbA1c, and FPG), and risk ratio (RR) with 95% CI for dichotomous outcomes (HbA1c reduction of ≥0.5%, hypoglycemia, ketone-related events and adverse events). Heterogeneity was evaluated using the I2 statistic, and I2 > 50% indicated significant heterogeneity^26^. The random-effects model used for all meta-analysis. We searched for potential sources of heterogeneity when encountering significant heterogeneity. Sensitivity analysis was performed to detect the influence of a single study on the overall estimate via omitting one study in turn or performing the subgroup analysis. Owing to the limited number (<10) of included studies, publication bias was not assessed. Results were considered as statistically significant for P <0.05. All statistical analyses were performed using Review Manager Version 5.3 (The Cochrane Collaboration, Software Update, Oxford, UK).

## Results

### Literature search, study characteristics and quality assessment

**Figure. 1:**
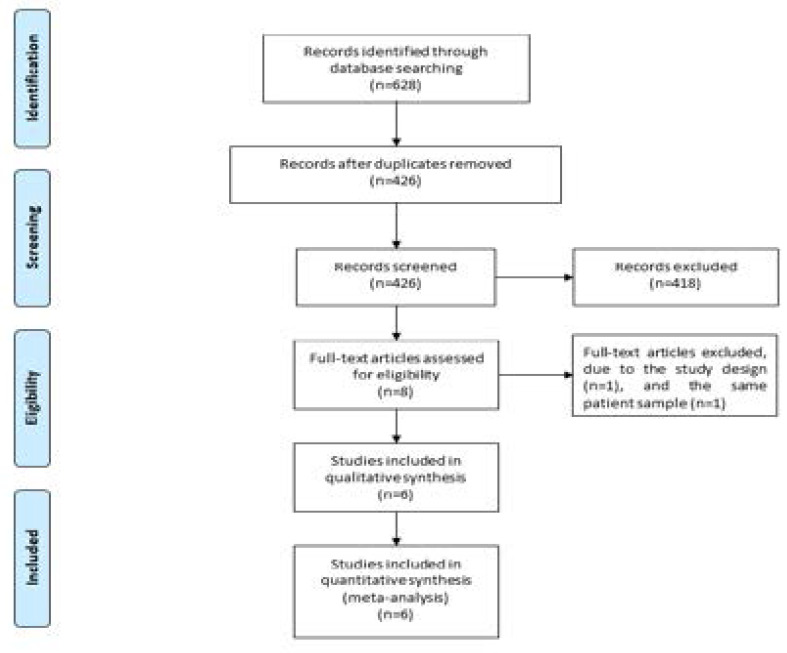
Flow diagram of study searching and selection process.

Figure 1 showed the detail flowchart of the search and selection results. 628 potentially relevant articles were identified initially. Finally, six RCTs were included in the meta-analysis 16–18, 21, 27, 28.

The baseline characteristics of six included RCTs were shown in Table 1. These studies were published between 2015 and 2018, and the total sample size was 1159. Two included RCTs reported the same patient sample at the follow-up of 24 weeks and 52 weeks 17, 20.

**Table 1:** Characteristics of included studies

NO.	Author	Dapagliflozin group						Control group						Jada scores
		Number	Age	Female (n)	BMI (kg/m2)	Duration of disease (year)	Methods	Number	Age	Female (n)	BMI (kg/m^2^)	Duration of disease (year)	Methods	
1	Watada 2018	14	37.1±10.2	7	22.2±2.1	14.7±12.4	dapagliflozin 10 mg once daily for 7 days	14	42.6±10.6	11	22.9±3.4	16.9±10.5	placebo	4
2	Melmer 2018	6	38.12±12.41	-	26.31±4.29	8.34±14.67	10 mg of dapagliflozin for 3 days	6	38.12±12.41 -		26.31±4.29	8.34±14.67	placebo	3
3	Mathieu 2018	270	42.4±12.80	149	27.80±5.53	19.45±11.90	dapagliflozin 10 mg once daily for 24 weeks	272	43.0±13.73	153	27.62±5.41	18.98±11.65	placebo	5
4	Dandona 2018	259	42.7±14.1	129	28.1±5.1	19.9±11.1	dapagliflozin 10 mg once daily for 52 weeks	260	42.7±13.6)	128	28.6±5.2)	21.2±12.2)	placebo	5
5	Kuhadiya 2016	20	55±3	10	31±1	25±3	dapagliflozin 10 mg daily for 12 weeks	10	52±3	6	27±2	31±5	placebo	5
6	Henry 2015	15	37.5±15.2	7	25.8±4.8	18.1±14.0	dapagliflozin 10 mg daily for 2 weeks	13	34.5±12.2	5	25.3±3.0	16.2±9.7	placebo	4

BMI = body mass index.

Among the six included RCTs, two RCTs reported HbA1c 17, 28, two RCTs reported HbA1c reduction of ≥0.5% 17, 18, two RCTs reported FPG 21, 28, four RCTs reported hypoglycemia 16–18, 28, three RCTs reported ketone-related events 17, 18, 28 and four RCTs reported adverse events 16–18, 21. Jadad scores of the six included studies varied from 3 to 5, and all six studies had high-quality based on the quality assessment.

### Primary outcomes: HbA1c and HbA1c reduction of ≥0.5%

**Figure. 2:**
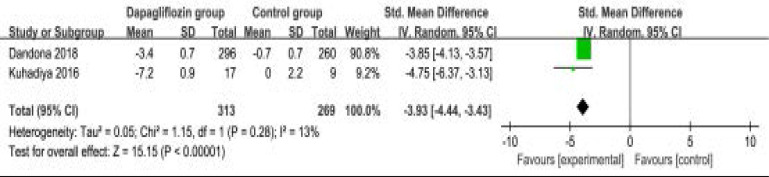
Forest plot for the meta-analysis of HbA1c.

**Figure. 3:**
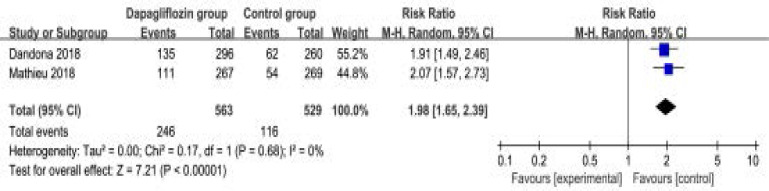
Forest plot for the meta-analysis of HbA1c reduction of ≥0.5%.

The random-effect model was used for the analysis of primary outcomes. The results revealed that compared to control group in patients with type 1 diabetes, dapagliflozin treatment could significantly reduce HbA1c (SMD=-3.93; 95% CI=-4.44 to -3.48; P<0.00001) with low heterogeneity among the studies (I2=13%, heterogeneity P=0.28, Figure 2) and improve HbA1c reduction of ≥0.5% (RR=1.98; 95% CI=1.65 to 2.39; P<0.00001 with no heterogeneity among the studies (I2=0%, heterogeneity P=0.68, Figure 3).

### Sensitivity analysis

There was low or no heterogeneity for the primary outcomes, and thus we did not perform the meta-analysis via omitting one study or subgroup analysis to detect the heterogeneity.

### Secondary outcomes

**Figure. 4:**
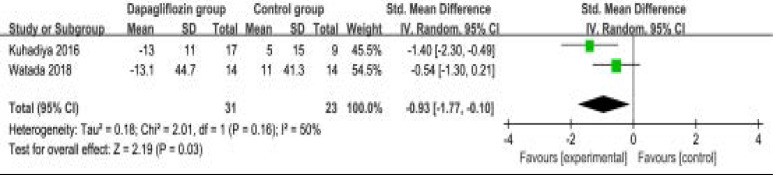
Forest plot for the meta-analysis of FPG.

**Figure. 5:**
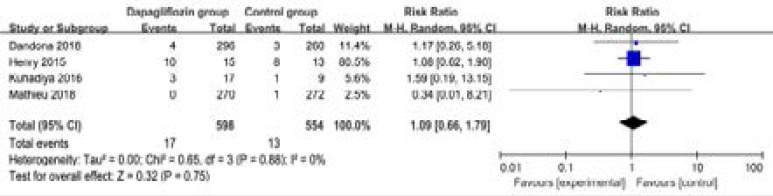
Forest plot for the meta-analysis of hypoglycemia.

**Figure. 6:**
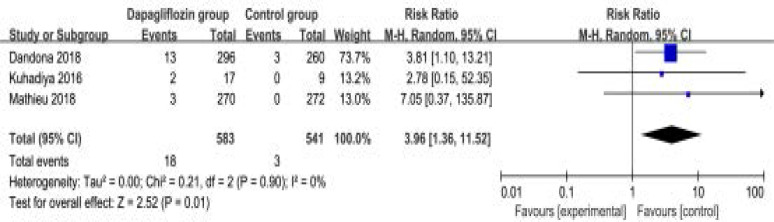
Forest plot for the meta-analysis of ketone-related events.

**Figure. 7:**
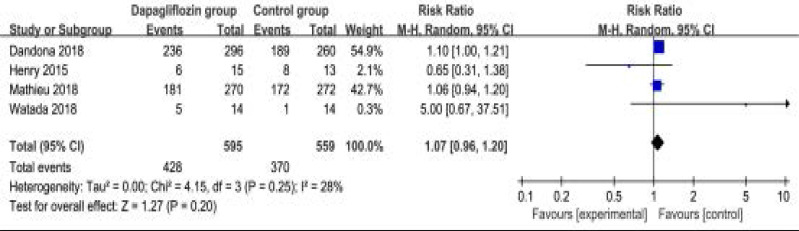
Forest plot for the meta-analysis of adverse events.

In comparison with control intervention for type 1 diabetes, dapagliflozin treatment was associated with the decrease in FPG (SMD=-0.93; 95% CI=-1.77 to -0.10; P=0.03; Figure 4), but showed no obvious impact on the incidence of hypoglycemia (RR=1.09; 95% CI=0.66 to 1.79; P=0.75; Figure 5). However, the incidence of ketone-related events was higher than those in control group (RR=0.28; 95% CI=3.96 to 11.52; P=0.01; Figure 6). There was no statistical difference of adverse events between two groups (RR=1.07; 95% CI=0.96 to 1.20; P=0.20; Figure 7).

## Discussion

SGLT2-inhibition has shown the efficacy for the treatment of type 1 and 2 diabetes. In patients with type 2 diabetes, dapagliflozin treatment was reported to increase muscle insulin sensitivity, and reduce endogenous glucose production and fasting glucagon 29. Several studies revealed that SGLT2-inhibition was able to reduce fasting and postprandial glucose concentrations, as well as HbA1c in type 1 diabetes 20, 30. In addition, dapagliflozin treatment resulted in insulin reduction of more than 30% in type 1 diabetes^21^.

Our meta-analysis suggests that dapagliflozin at the dose of 10 mg daily shows the significant benefits in HbA1c, HbA1c reduction of ≥0.5% and FPG in patients with type 1 diabetes. Furthermore, treatment with dapagliflozin 5 mg and 10 mg as adjunct therapy to insulin may improve glycemic control in these patients and reduce insulin dose by −19.3% (95% CI, −30.1 to −6.8) and − 16.2% (95% CI, −29.4 to −0.5), respectively 16. There is no significant difference of hypoglycemia and adverse events between two groups based on the results of this meta-analysis. These adverse events are well tolerated.

However, our meta-analysis reveals that dapagliflozin treatment appears to increase the ketone-related events compared to placebo. In DEPICT-2 study, the incidence of diabetic ketoacidosis was reported to be higher compared with DEPICT-1 study (dapagliflozin 5 mg versus dapagliflozin 10 mg versus placebo: 5.83, 4.99, and 0 per 100 patient-years in DEPICT-2, respectively; 3.29, 3.78, and 2.64 per 100 patient-years in DEPICT-1) 17, 18, 20. The variability may be caused by the small number of events, and different risk factors to develop diabetic ketoacidosis. The events of diabetic ketoacidosis were generally resolved using conventional treatments 18

Several limitations exist in this meta-analysis. Firstly, our analysis is based on only six RCTs, and more RCTs with large samplsize should be conducted to explore this issue. Although there is low heterogeneity, different treatment duration of dapagliflozin may lead to some bias. Finally, some unpublished and missing data may lead to some bias to the pooled effect.

## Conclusion

Dapagliflozin treatment can provide some benefits for glycemic control in patients with type 1 diabetes, as evidenced by the reduction in HbA1c and FPG.
